# Comparing manual vs. automated machine learning and deep learning models for predicting one-year mortality in elderly hip fracture patients

**DOI:** 10.3389/fmed.2026.1804645

**Published:** 2026-06-01

**Authors:** Adi Shuchami, Maxim Glebov, Maksim Katsin, Yotam Portnoy, Haim Berkenstadt, Dina Orkin, Teddy Lazebnik

**Affiliations:** 1 Department of Mathematics, Ariel University, Ariel, Israel; 2 Department of Anesthesiology, Sheba Medical Center, Ramat Gan, Israel; 3 Department of Information Systems, University of Haifa, Haifa, Israel; 4 Faculty of Medicine, Tel-Aviv University, Tel Aviv, Israel; 5 Department of Computing, Jönköping University, Jönköping, Sweden

**Keywords:** automatic machine learning, clinical decision support, large language model, logistic regression, autoML

## Abstract

**Background:**

Hip fractures are associated with significant mortality, especially among elderly patients. Accurate prediction of mortality risk is crucial for optimising perioperative care and resource allocation. Recent advances in machine learning (ML) and deep learning (DL) offer promising methods to enhance clinical risk prediction models; however, their clinical implementation often remains limited due to the complexity of these techniques.

**Methods:**

This retrospective cohort study included 2,604 elderly patients (≥65 years) undergoing urgent hip fracture surgery at Sheba Medical Center, Israel, between January 2017 and November 2023. Multiple ML and DL algorithms were evaluated for predicting one-year all-cause mortality using a comprehensive set of clinical, demographic, perioperative, and laboratory variables. Models were rigorously developed and validated through stratified 5-fold cross-validation, addressing class imbalance with the Synthetic Minority Oversampling Technique (SMOTE). Additionally, an automated ML pipeline, generated using a large language model (LLM) coupled with the Tree-based Pipeline Optimisation Tool (TPOT), was benchmarked against manually optimised models. Model performances were assessed using area under the receiver operating characteristic curve (AUC), accuracy, precision, recall, F1-score, false-positive rate, and true-negative rate, supplemented by permutation importance and SHapley Additive exPlanations (SHAP) for interpretability.

**Results:**

Among all models evaluated, the manually optimised Extreme Gradient Boosting (XGB) algorithm demonstrated superior predictive performance (AUC = 0.846, accuracy = 0.791, F1-score = 0.667, precision = 0.773, NPV = 0.798). Important predictors identified included baseline serum albumin and urea levels, patient age, intraoperative hypothermia, and the number of chronic diseases. The automated ML model, generated via LLM and TPOT frameworks, showed comparable performance to the XGB model (AUC = 0.844), with a higher recall but slightly lower precision.

**Discussion:**

ML-based models, particularly the XGB algorithm, significantly enhance predictive accuracy for one-year mortality among elderly hip fracture patients. Crucially, an automated ML framework leveraging large language models provides a practical, clinically accessible alternative, effectively democratising advanced predictive analytics in healthcare settings.

## Introduction

Hip fracture represents a significant global health concern, with projections estimating approximately 4.5 million annual cases worldwide by 2050 (1). This injury predominantly affects older adults, leading to substantial clinical and economic burdens on healthcare systems globally (2). Postoperative mortality rates remain alarmingly high, with studies reporting up to 36% mortality within the first year following surgical intervention (3). These concerning figures highlight an urgent need for accurate risk stratification tools capable of identifying high-risk patients and guiding perioperative management, ultimately aiming to reduce preventable mortality.

Advancements in data-driven approaches, particularly machine learning (ML), have enabled the development of sophisticated clinical prediction models integrating perioperative and broader clinical variables (4). Unlike traditional regression techniques, ML algorithms effectively manage real-world data characterized by complex, nonlinear predictor interactions, often resulting in superior predictive accuracy (5). Indeed, comparative studies assessing ML-based models against conventional statistical approaches in predicting hip fracture mortality have consistently demonstrated enhanced performance, reinforcing the rationale for further exploration and adoption of ML methodologies (6–8).

This study aims to evaluate various ML and deep learning (DL) algorithms for predicting one-year mortality among elderly hip fracture patients. Crucially, we investigate the effectiveness of an automated ML platform facilitated by a large language model (LLM) in empowering clinicians with limited technical expertise to independently develop robust and clinically meaningful prediction models. By specifically addressing this practical aspect, our study underscores the potential for automated ML platforms to significantly democratize access to advanced predictive analytics in clinical settings. We aim to benchmark the performance of these automated ML and DL approaches against established predictive models, thereby demonstrating the added value and practicality of such frameworks for enhancing clinical risk prediction and informed decision-making.

## Materials and methods

### Ethics approval and reporting guidelines

This study received ethical approval from the Institutional Ethics Committee at Sheba Medical Center, Israel (Approval No. SMC-D 0976-24), and was performed in accordance with the relevant guidelines and regulations in accordance with the Declaration of Helsinki. Due to the retrospective observational design of the study, the requirement for informed consent was waived by the committee. The study protocol and reporting follow the Transparent Reporting of a Multivariable Prediction Model for Individual Prognosis or Diagnosis with Artificial Intelligence (TRIPOD-AI) guidelines.

### Study design and population

This retrospective cohort study was conducted at a single tertiary care center (Sheba Medical Center, Israel). The study population included elderly patients, aged 65 years and older, undergoing primary emergency surgery for hip fracture between 1 January 2017 and 1 November 2023. Data extraction was performed using electronic health records from the institution’s database system (Chameleon, Elad Software Ltd., Tel Aviv, Israel). Eligible cases were identified through International Classification of Diseases, Tenth Revision (ICD-10) diagnosis codes: S72.0 (Fracture of neck of femur), S72.1 (Pertrochanteric fracture), and S72.2 (Subtrochanteric fracture). Patients were included if they had complete clinical data and a minimum follow-up of 1 year or until death. Exclusion criteria encompassed high-energy trauma mechanisms, multiple fractures, open fractures, and pathological fractures secondary to malignancy or metabolic bone disease.

### Data collection

Data collected encompassed a comprehensive set of variables, including demographic characteristics, functional and social assessments, comorbidities, laboratory parameters, physiological measurements, intraoperative details, and postoperative outcomes. A detailed description of variables is provided in Supplementary Table 1.

The dataset was extracted from the hospital electronic medical record (EMR) environment, where data were already organized within a structured clinical data framework designed to support harmonization, formatting consistency, and routine data quality control. In accordance with best practices for clinical ML preprocessing, the dataset was reviewed to confirm variable definitions, preserve clinically appropriate coding, and ensure that the exported table was analysis-ready for supervised learning. Because the data originated from an EMR system with standardized organization, major preprocessing focused primarily on verification of completeness, consistency, and correct variable representation rather than large-scale manual restructuring. Subject-matter expertise remained essential at this stage to confirm the clinical meaning of variables, ensure that preprocessing decisions were medically appropriate, and verify that the final dataset accurately reflected real-world perioperative and baseline patient information. These preprocessing steps are important for both modeling strategies presented in this study to ensure clinical relevance and usability.

### Sample size calculation

Given the relatively balanced nature of our dataset, we included a 20% contingency in our initial sample size estimation to account for potential undersampling and to maintain data balance. Considering the complexity of the predictive model and the total number of features, we adhered to the established methodological recommendation of at least 10 events per variable (EPV) (9). Since our dataset includes 98 variables, the minimum sample size required is 1,176 patients.

### Outcome

The primary outcome was all-cause one-year mortality, defined as death occurring within 1 year from the date of fracture. Mortality data were obtained from the Ministry of Health’s vital status registry, and accuracy was subsequently validated by manual review of patient medical records.

### Model development: manual model development and evaluation

**Figure 1:**
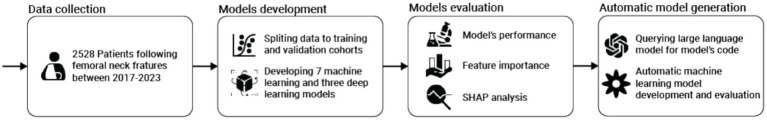
A schematic view of the methodological framework of this study.

Figure 1 illustrates a schematic representation of the methodological framework employed in this study.

Initially, descriptive statistical analyses were conducted to characterize the dataset. Subsequently, a Pearson correlation matrix was generated to investigate linear associations among features and their correlation with one-year mortality.

To develop machine learning (ML) and deep learning (DL) predictive models, the dataset was partitioned into training (80%) and validation cohorts (20%). The training cohort was further subjected to *k*-fold cross-validation (10) to ensure robust model evaluation. In this process, the data were divided into five equally sized, mutually exclusive subsets. Each subset served as the validation cohort exactly once, while the remaining subsets formed the training set, resulting in five validation iterations. Model performance was averaged across iterations to estimate predictive capability reliably.

Both training and validation cohorts were stratified by age and sex to ensure representative distributions. Within each cross-validation fold, an optimisation process was employed to minimise demographic differences in age and sex distributions across subsets. This optimisation, analogous to the nurse scheduling problem (11)—an NP-hard computational problem (12)—was addressed using Directed Bee Colony Optimisation to achieve a near-optimal solution (13).

Following data partitioning, seven ML models and three DL models were developed and comparatively evaluated. ML algorithms included logistic regression (LR) (14), naïve Bayes (NB) (15), k-nearest neighbours (kNN) (16), support vector machine (SVM) (17), decision tree (DT) (18), random forest (RF) (19), and XGBoost (XGB) with $L_{2}$ regularization (20). DL algorithms comprised multilayer perceptron (MLP) (21), TabNet (22), and a transformer-based tabular model (TabTransformer, TT) (23). Hyperparameter tuning was performed via grid search (24), systematically testing parameter combinations to optimise model accuracy during cross-validation.

Because the primary outcome was moderately imbalanced (one-year mortality rate: 36.9%), we evaluated each model under two settings: non-balanced, in which models were trained using the original class distribution, and balanced, in which Synthetic Minority Oversampling Technique (SMOTE) (25) was applied to the training folds to generate a more even class distribution. The validation/test data were kept unchanged to preserve a realistic clinical evaluation setting. This comparison allowed us to assess how class balancing influences predictive performance, particularly the trade-off between sensitivity to mortality events and false-positive predictions. This comparison provided insights into the impact of data balancing on model accuracy and feature importance.

Model performance in the validation cohort was evaluated comprehensively using multiple metrics: accuracy, F1-score, recall (sensitivity), precision, false positive rate (FPR), true negative rate (TNR), negative predictive values (NPV), and the area under the receiver operating characteristic (ROC) curve (AUC). Feature importance for each model was assessed using permutation importance (26), quantifying how random permutation of each feature influenced model performance. Additionally, SHapley Additive exPlanations (SHAP) (27) were utilised to offer detailed, instance-level insights into individual feature contributions to model predictions. All analyses are performed using the Python programming language.

### Model development: automatic model development and evaluation

As the analytical procedures outlined above require advanced expertise in ML and DL, or at least significant experience in data-driven model development, they remain challenging and largely inaccessible for most clinicians. However, recent advancements in automated ML frameworks, coupled with the wider availability of LLMs such as ChatGPT (28), have prompted exploration into whether these tools could facilitate the creation of “out-of-the-box” ML models. Specifically, we aimed to determine whether such automated approaches could generate a viable predictive model, evaluate its performance rigorously, and present results clearly and understandably to clinical professionals without specialized ML knowledge.

To investigate this, we provided the ChatGPT-o3 model (29) with a structured prompt (Supplemental material A). The model-generated Python code was executed directly, without modification (“as-is”), in a Google Colab environment, and the results obtained from this automated approach are presented below.

The structured prompt was carefully designed to replicate the analytical methodology applied previously in our manually developed models. For the automated ML component, we chose the Tree-based Pipeline Optimization Tool (TPOT) (30) due to its extensive prior application (31) and proven reliability in clinical prediction tasks (32). The prompt included a definition of the LLM’s “persona”—a method previously demonstrated to enhance LLM performance (33)—and concluded with explicit, directive instructions detailing the desired analytical outcomes.

Predictive performance of the automated ML-generated model was rigorously assessed using a comprehensive set of metrics identical to those employed for manual model evaluations. Additionally, we analysed feature importance and applied SHAP to achieve an in-depth, instance-level interpretation of each feature’s contribution to the model’s predictions.

## Results

### Descriptive statistics

The final cohort included 2,604 patients, of whom 1719 (66.0%) were female. The mean patient age was 82.3 years (SD 8.3). Detailed demographic and baseline characteristics of the cohort are summarised in Table 1.

**Table 1:** Demographic and baseline characteristics of the cohort.

Characteristic	All cohort
Age (years)	82.3 + −8.26
Female	66.0%
BMI (kg/m^2^)	25.08 + −4.29
Surgery duration (min)	55 + −31
PACU time (min)	108 + −52
Previous hospitalization in 6 months	25.6%
Fracture to surgery time less than 48 h	87.1%

All data are presented as the mean [SD] or *n* (%). BMI, Body mass index; PACU, Post-anesthesia care unit.

**Figure 2:**
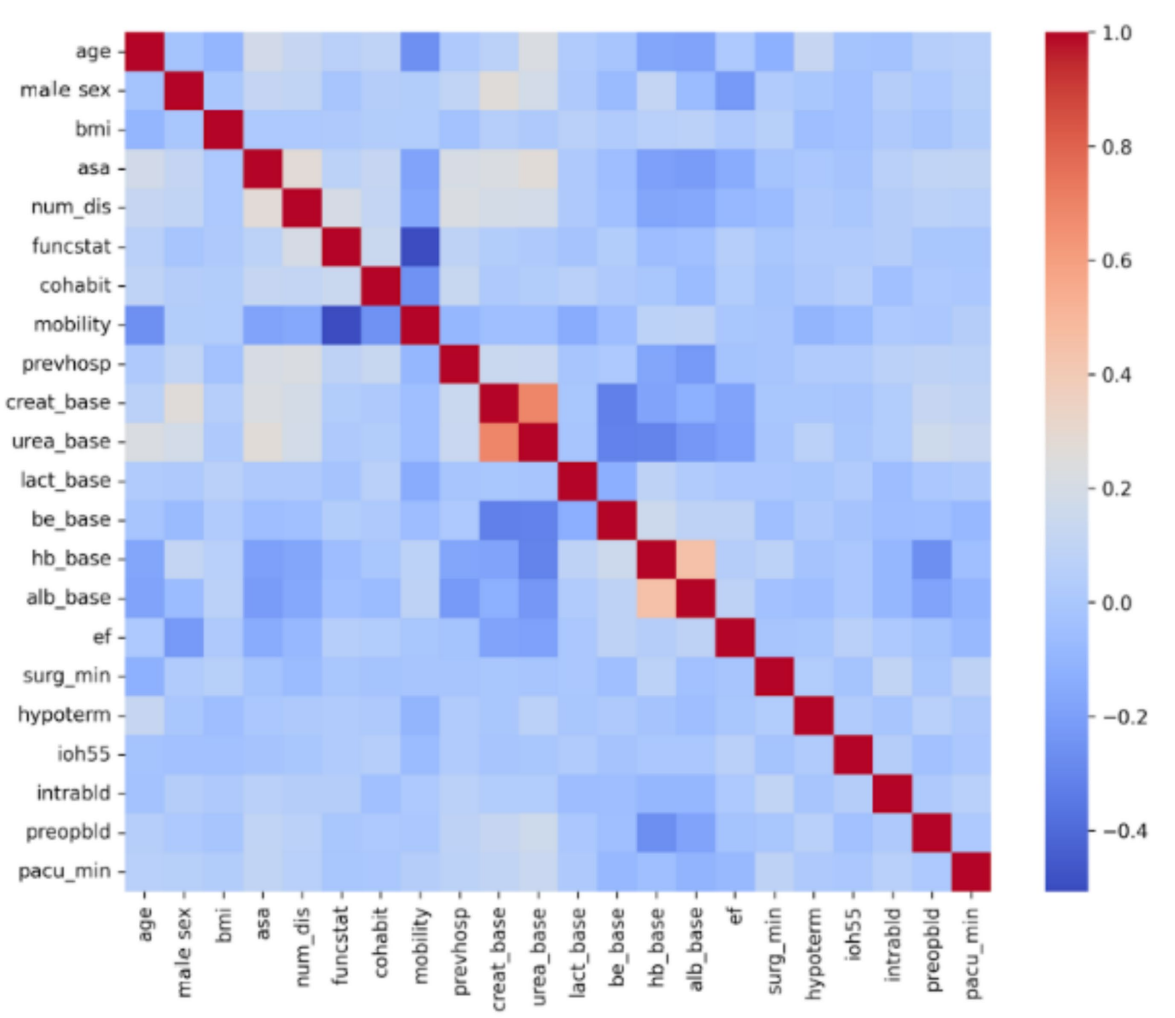
Pearson correlations between the dataset’s features. BMI, body mass index; ASA, American Society of Anesthesiologists Physical Status classification; num_dis, number of diseases; funcstat, functional status; cohabit, cohabitation status; mobility, mobility status; prevhosp., previous hospitalisation within 6 months; creat_base, baseline creatinine; urea_base, baseline urea; lact_base, baseline lactate; be_base, baseline base excess; hb_base, baseline haemoglobin; alb_base, baseline albumin; EF, ejection fraction; surg_min, surgery duration in minutes; hypoterm, intraoperative hypothermia; ioh55, intraoperative hypotension (MAP<55 mmHg); intrabld, intraoperative blood transfusion; preopbld, preoperative blood transfusion; pacu_min, duration in post-anaesthesia care unit in minutes.

Figure 2 displays the Pearson correlation matrix for the variables incorporated into the predictive model and the target outcomes. Most predictors showed minimal intercorrelation, with correlation coefficients approaching zero. More importantly, as indicated by the last row, the source features are poorly linearly correlated to the target variable. Therefore, a nonlinear machine learning method is anticipated to demonstrate superior predictive performance compared with traditional linear approaches such as logistic regression. Notably, due to the symmetric nature of correlations, the values above and below the main diagonal line are identical.

### Model performance

**Figure 3:**
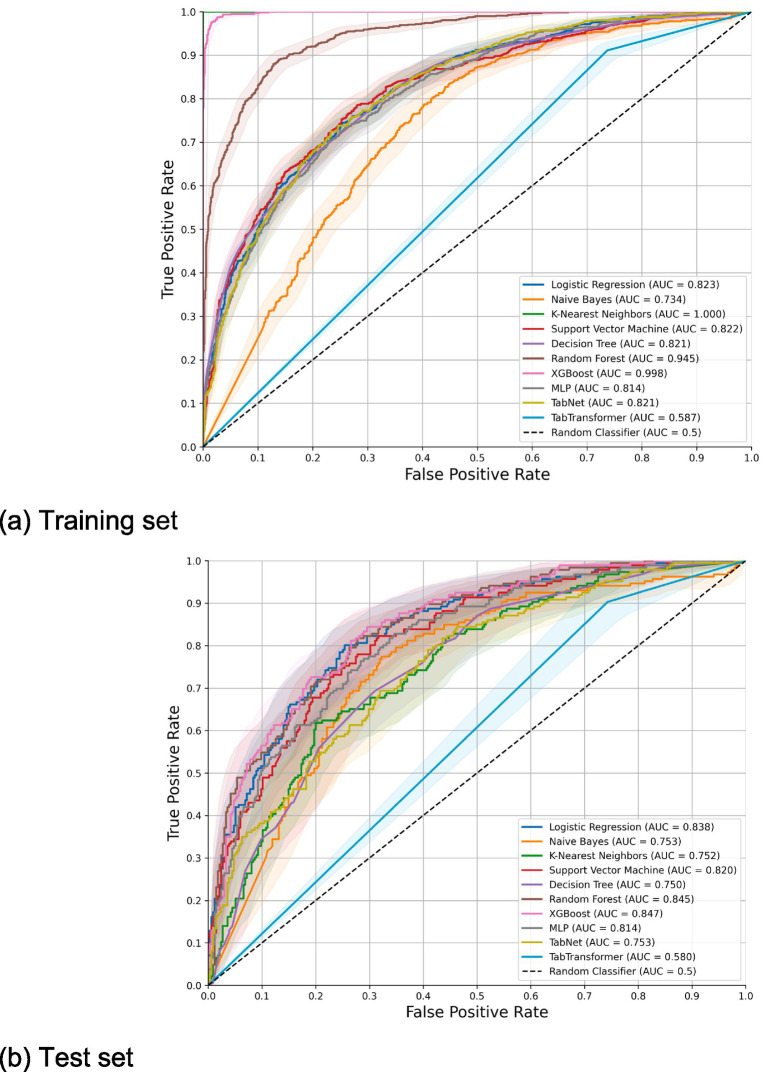
The ROC curves illustrate the performance of all ten models in the training cohort. Shaded areas represent the 95% confidence intervals derived from the k-fold (k = 5) cross-validation splits. **(a)** Training set; **(b)** Test set.

Figure 3 demonstrates the receiver operating characteristic (ROC) curves for all ten evaluated models. The results from the training cohort are presented with corresponding 95% confidence intervals (CIs), calculated using five-fold cross-validation. Overall, the models exhibited comparable discriminatory abilities, although the K-Nearest Neighbour (KNN), TabNet, and Naïve Bayes (NB) algorithms consistently demonstrated lower area under the curve (AUC) values compared to the remaining models across both training and testing datasets. Of particular note, the TT model exhibited the lowest performance, with an AUC of 0.580, suggesting possible underfitting. To provide a more comprehensive evaluation, we further analysed additional relevant performance metrics. Importantly, the figure presents the ROC curves for models trained on the original, non-resampled dataset, in order to compare discrimination under the observed class distribution.

Table 2 summarises the performance metrics of ML and DL models on the test dataset, including area under the receiver operating characteristic curve (AUC), accuracy, F1 score, recall, precision, false-positive rate (FPR), true-negative rate (TNR), and negative predictive value (NPV). In the slightly imbalanced scenario, with a one-year mortality rate of 36.9%, the extreme gradient boosting (XGB) model demonstrated superior performance, achieving the highest AUC (0.846), accuracy (0.791), and F1 score (0.667). The Naive Bayes (NB) model exhibited the highest recall (0.968) but had the lowest accuracy (0.380). Although the DL-based TabTransformer (TT) model showed excellent precision (0.857), FPR (0.003), and TNR (0.997), its low recall (0.032) and modest AUC (0.580) suggest a bias towards predicting a single class, thus significantly restricting its clinical applicability.

**Table 2:** Performance of ML and DL models on the test dataset.

Balanced	Model	AUC	Accuracy	F1 score	Recall	Precision (PPV)	FPR	TNR	NPV
No	LR	0.838	0.766	0.628	0.554	0.725	0.116	0.884	0.781
	NB	0.753	0.380	0.527	**0.968**	0.362	0.946	0.054	0.753
	KNN	0.752	0.693	0.416	0.306	0.648	0.093	0.907	0.703
	SVM	0.820	0.750	0.622	0.575	0.677	0.152	0.848	0.781
	DT	0.750	0.697	0.503	0.430	0.606	0.155	0.845	0.728
	RF	0.845	0.754	0.552	0.425	0.790	0.063	0.937	0.746
	XGB	**0.846**	**0.791**	**0.667**	0.586	0.773	0.096	0.904	**0.798**
	MLP	0.823	0.758	0.627	0.57	0.697	0.137	0.863	0.782
	Tabnet	0.707	0.655	0.118	0.065	0.667	0.018	0.982	0.655
	TT	0.580	0.653	0.062	0.032	**0.857**	**0.003**	**0.997**	0.650
Yes	LR	0.827	0.760	**0.694**	0.763	0.637	0.242	0.758	0.828
	NB	0.748	0.376	0.524	**0.962**	0.360	0.949	0.051	0.708
	KNN	0.646	0.597	0.551	0.694	0.457	0.457	0.543	0.761
	SVM	0.743	0.678	0.538	0.527	0.551	0.239	0.761	0.745
	DT	0.601	0.637	0.538	0.591	0.493	0.337	0.663	0.741
	RF	**0.849**	0.772	0.674	0.720	0.667	0.200	0.800	**0.841**
	XGB	0.833	**0.775**	0.693	0.651	**0.699**	**0.155**	**0.845**	0.811
	MLP	0.765	0.718	0.608	0.613	0.603	0.224	0.776	0.784
	Tabnet	0.753	0.658	0.615	0.763	0.514	0.400	0.600	0.821
	TT	0.503	0.488	0.557	0.903	0.403	0.743	0.257	0.826

ML, machine learning; DL, deep learning; LR, logistic regression; NB, naïve Bayes; KNN, k-nearest neighbours; SVM, support vector machine; DT, decision tree; RF, random forest; XGB, XGBoost; MLP, multi-layer perceptron; TT, TabTransformer; AUC, area under the receiver operating characteristic curve; FPR, false positive rate; TNR, true negative rate. Best-performing values for each metric are highlighted in bold. The balanced group indicates models trained using SMOTE-balanced data, while the other group indicates models trained on the original, non-resampled data.

In the balanced scenario, a similar performance pattern emerged, with XGB consistently outperforming other models across most metrics. However, the random forest (RF) model achieved a slightly higher AUC (0.849), and the NB model exhibited superior recall (0.962), surpassing XGB by absolute margins of 0.016 and 0.311, respectively. Nonetheless, NB continued to demonstrate substantially lower accuracy (0.376) compared to XGB (0.775). Overall, when considering all performance metrics comprehensively, the XGB model emerged as clearly superior and was consequently selected for subsequent analyses. Specifically, XGB is well-suited to the structured clinical dataset and also achieved the best F1 score, which considered a key metric because it captures the balance between recall and precision in an imbalanced mortality prediction task. From a clinical perspective, recall is especially important, since failing to identify a patient at high risk of one-year mortality may result in missed opportunities for closer follow-up, earlier intervention, or more intensive care planning. However, focusing on recall alone may favor models that label too many patients as high risk, thereby reducing precision. In practice, this can create a biased prediction pattern that overestimates risk and generates unnecessary clinical workload, including excess alerts, avoidable evaluations, and inefficient use of healthcare resources.

### Feature importance

**Figure 4:**
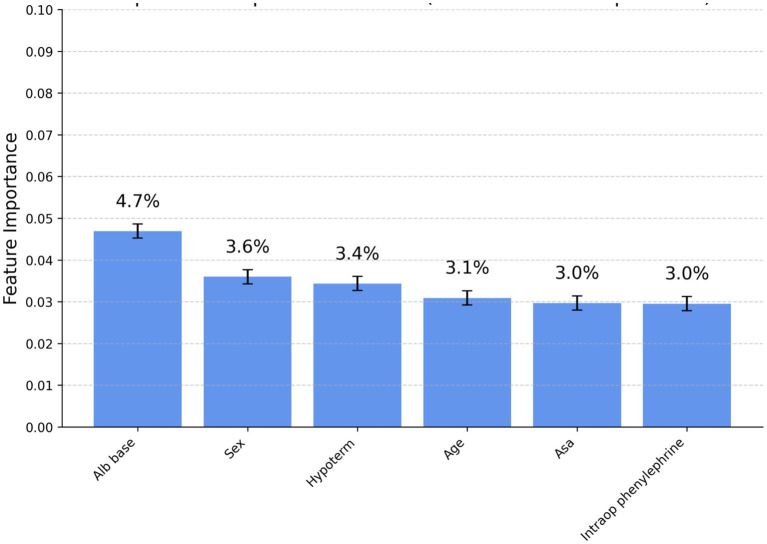
Feature importance distribution for the XGBoost prediction model. alb_base, baseline albumin; hypoterm, intraoperative hypothermia; ASA, American Society of Anesthesiologists Physical Status classification; intraop phenylephrine, intraoperative use of phenylephrine.

For interpretability analysis, we focused on the XGB model trained on the balanced dataset, as this version was selected for subsequent detailed analysis after comparison of balanced and non-balanced training. To this end, Figure 4 illustrates the distribution of feature importance derived from the XGB model trained on the balanced dataset. Due to the extensive number of variables, only the six most influential features are presented. The most significant predictors identified were baseline albumin, baseline urea, and patient age.

**Figure 5:**
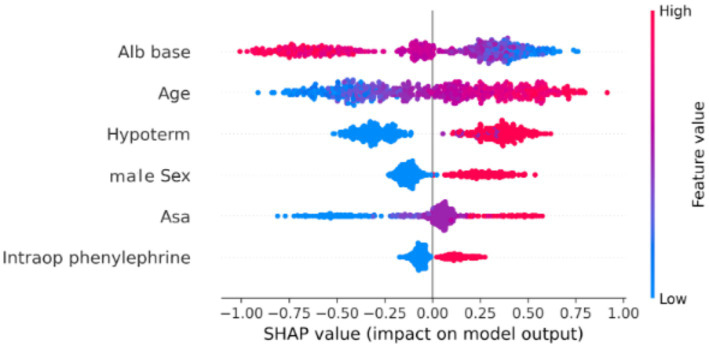
SHAP analysis of the XGB model. alb_base, baseline albumin; hypoterm, intraoperative hypothermia; ASA, American Society of Anesthesiologists Physical Status classification; intraop phenylephrine, intraoperative use of phenylephrine.

Figure 5 illustrates the SHAP analysis for the XGB model where each dot represents one individual patient/sample. The x-axis shows the SHAP value, indicating the direction and magnitude of that feature’s contribution to the predicted one-year mortality risk. Dot color reflects the original feature value (low to high). Features are ordered according to their overall importance in the model. This analysis identifies patient age as the most influential predictor, with older age strongly associated with an increased likelihood of one-year mortality. Baseline serum albumin, the second most significant feature, indicates that lower albumin levels correlate with elevated mortality risk. A comparable association is observed for intraoperative hypothermia, ranked as the third most impactful feature. Additionally, male sex is identified as a predictor associated with higher mortality rates. Finally, the American Society of Anesthesiologists (ASA) score demonstrates a monotonic relationship with the predicted probability of mortality within one year.

### Automatic ML model

In comparison, the automated ML framework, generated through code written by an LLM, identified an ensemble majority-vote model comprising CatBoost, kNN, and RF as the optimal solution for the given dataset. Specifically, the final prediction of this automated model reflects the consensus reached by at least two of the three constituent algorithms. The code for this automated framework, generated by the LLM, as well as the code for the model derived using the TPOT framework, is provided in Supplementary material B.

Table 3 presents a comparative analysis of the predictive performance between the automatically generated models (LLM and TPOT) and the manually optimized best-performing model (XGB). Overall, predictive performance metrics between the automated and manually developed models were comparable, with accuracy showing the largest discrepancy. Differences in area under the receiver operating characteristic curve (AUC) between both models were negligible in both balanced and unbalanced data scenarios (0.002 and 0.012, respectively). The manually developed XGB model exhibited superior precision, false positive rate (FPR), and true negative rate (TNR). Conversely, the automated ML model demonstrated superior performance in terms of the F1 score and recall metrics.

**Table 3:** Performance comparison between the automatically derived model (LLM and TPOT) and the manually developed best-performing model (XGB).

**Balanced**	**Model**	**AUC**	**Accuracy**	**F1 score**	**Recall**	**Precision**	**FPR**	**TNR**
No	XGB	**0.846**	**0.791**	0.667	0.586	**0.773**	**0.096**	**0.904**
	LLM and TPOT	0.844	0.726	**0.698**	**0.887**	0.575	0.364	0.636
Yes	XGB	**0.833**	**0.775**	0.693	0.651	**0.699**	**0.155**	**0.845**
	LLM and TPOT	0.825	0.718	**0.720**	**0.891**	0.605	0.129	0.833

LLM, large language model; TPOT, Tree-based Pipeline Optimization Tool; XGB, XGBoost; AUC, area under the receiver operating characteristic curve; FPR, false positive rate; TNR, true negative rate. The best-performing values for each metric are highlighted in bold.

**Figure 6:**
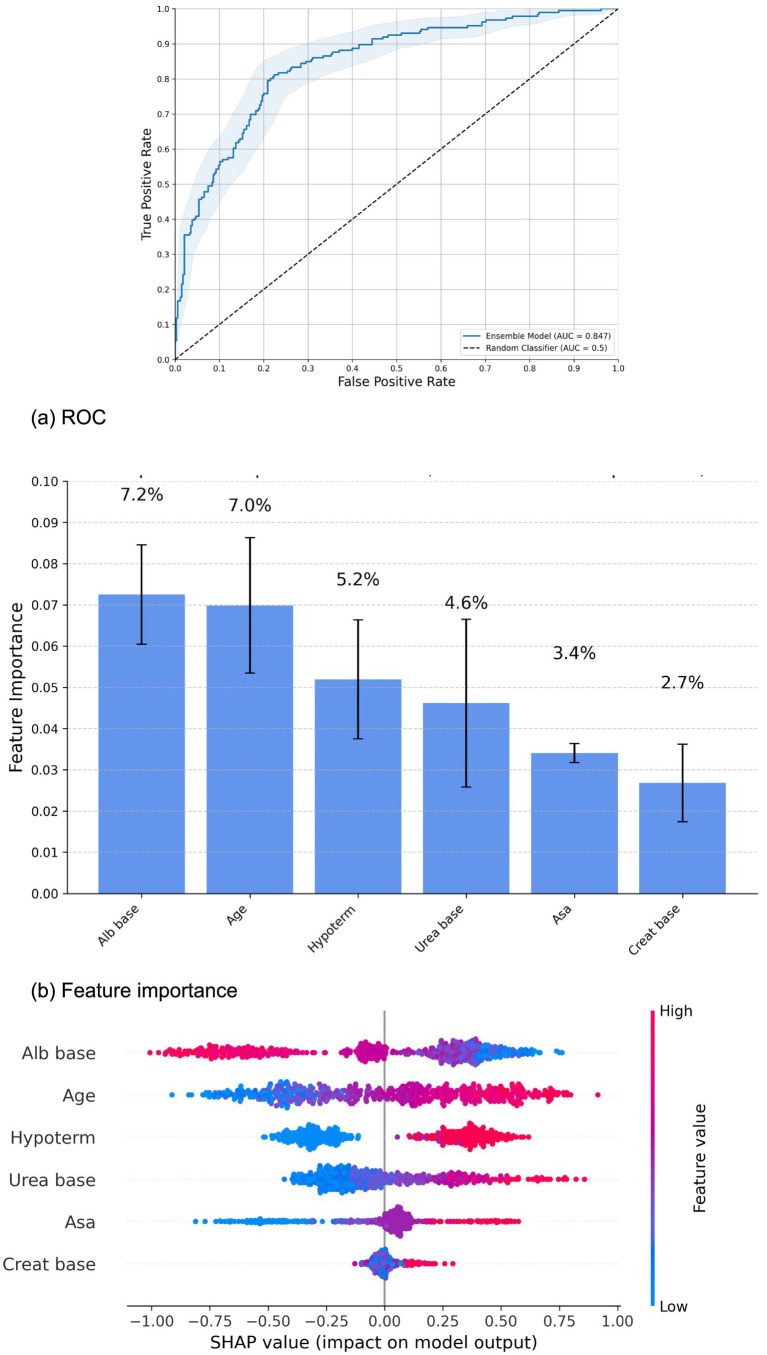
Performance evaluation and explainability analysis of the predictive model developed using the LLM and automated ML framework. **(a)** ROC; **(b)** Feature importance; **(c)** SHAP analysis. Abbreviations: alb_base, baseline albumin; hypoterm, intraoperative hypothermia; urea base, baseline urea; ASA, American Society of Anesthesiologists Physical Status classification; create base, baseline creatinine.

Figures 6a–c presents the ROC curve, feature importance, and SHAP analysis for the model automatically derived using the LLM and the automated machine learning framework (TPOT). The ROC curve demonstrates performance comparable to the XGB model. The feature importance analysis reveals substantial overlap with the top six features identified by the XGB model, albeit with minor variations in ranking. Similarly, SHAP analysis confirms this concordance, highlighting consistent clinical interpretability across both models.

## Discussion

In this study, we systematically evaluated the potential of ML and DL models to support clinical decision-making by predicting one-year mortality in elderly patients with hip fractures. Our findings indicate that traditional ML methods generally outperform DL models when applied to structured tabular clinical data, a result consistent with recent comprehensive evaluations conducted across diverse clinical datasets (31, 34).

Among the various predictive models assessed, the manually developed XGB model demonstrated the highest predictive accuracy, thereby offering clinicians a robust and reliable tool to effectively identify high-risk patients. This finding aligns closely with previous clinical investigations utilising machine learning approaches, which consistently report superior performance of XGB in a variety of clinical prediction tasks due to its capacity to handle complex, high-dimensional data efficiently (35). Specifically, XGB was favored not only because of its overall predictive performance, but also because it achieved the best F1 score as indicated in Table 2. Such predictive accuracy has profound clinical implications, as early and precise identification of high-risk patients allows clinicians to implement timely interventions, potentially improving survival rates, reducing morbidity, and enhancing overall patient outcomes.

The strength of the XGB model primarily resides in its capacity to integrate multiple, heterogeneous clinical predictors, effectively mirroring the intricacies of real-world clinical practice. Key predictors identified by our analysis; such as patient age (36), baseline serum albumin (37), baseline serum urea (38), and intraoperative hypothermia (39), represent well-established clinical risk factors previously corroborated in the literature. This consistency significantly reinforces the clinical validity and interpretability of our findings (6). Furthermore, by quantifying the relative contributions of these predictors through advanced feature importance metrics and SHAP analyses, our study not only reinforces existing clinical knowledge but also provides clinicians with actionable insights into patient-specific risk profiles. Consequently, this facilitates targeted resource allocation, optimised care delivery, and prioritisation of interventions tailored to those patients identified as being at the highest risk.

Remarkably, we also explored the innovative approach of leveraging a large language model (ChatGPT-o3) to automatically generate ML code within the TPOT automated machine learning framework. The predictive performance of this automatically generated model was comparable, albeit slightly inferior, to that of the manually optimised XGB model. This similarity in both predictive performance and clinical interpretability underscores the promising utility of automated ML workflows, particularly for clinicians and institutions lacking extensive technical expertise in data science. Such an automated approach offers the distinct advantage of rapidly developing predictive analytics, facilitating continual model updating, refinement, and retraining as new clinical data becomes available, thus maintaining clinical applicability over time. Accordingly, the key difference between the two approaches is not the specific presence or absence of individual algorithms, but whether pipeline construction, model combination, and selection were performed manually by the research team or automatically by the LLM/TPOT workflow.

Importantly, in real clinical practice, both the manual ML workflow and the automated LAG/LLM-based workflow would require a human-in-the-loop. In the manual workflow, this would likely involve data scientists or clinicians with ML expertise performing dataset preparation, feature handling, model selection, tuning, and validation before the model could be implemented in a clinical decision-support setting. In the automated LLM-based workflow, expert oversight would still be necessary to formulate the clinical question, verify the suitability of the dataset, review the generated pipeline, and assess the clinical plausibility and safety of the resulting model (40). Thus, the difference between the two approaches is not the elimination of human involvement, but rather the degree of technical burden and specialization required. The automated approach may reduce the need for advanced hands-on ML expertise and shorten development time, whereas the manual workflow is generally more resource-intensive and dependent on dedicated methodological expertise. Accordingly, the automated workflow may be better viewed as a tool for reducing development barriers and resource demands, rather than as a fully autonomous replacement for expert-driven model development (41, 42).

From a practical and operational standpoint, the predictive models developed and validated in this study can be seamlessly integrated into existing clinical infrastructures, such as electronic health record systems, enabling real-time risk assessment at the point of care. Integrating these predictive analytics into clinical workflows could significantly enhance postoperative management strategies, guide rehabilitation planning, and optimise resource allocation decisions. The timely identification of high-risk elderly patients undergoing hip fracture surgery could prompt targeted interventions, intensified monitoring, proactive rehabilitation strategies, and personalised follow-up plans, potentially leading to improved clinical outcomes and enhanced patient satisfaction.

Nevertheless, the findings from our study must be cautiously interpreted within the scope of several inherent limitations. First, our patient cohort was exclusively derived from a single institution, potentially limiting generalisability and introducing institution-specific biases. Second, our analysis was confined to static, routinely available clinical variables, excluding potentially informative dynamic variables such as serial vital signs, evolving laboratory markers, or longitudinal patient status data, which may enhance predictive performance when integrated. Third, our dataset represents a relatively limited timeframe, and evolving treatment paradigms, changes in clinical practice, or shifting patient demographics could influence future model performance. Consequently, periodic updating, external validation in independent patient populations, and longitudinal model recalibration are essential for ensuring continued clinical relevance and robustness. Finally, an additional consideration is that the present model was developed for all-cause one-year mortality, which represents a broad binary endpoint. If sufficiently detailed and reliable cause-of-death data were available, future studies could examine whether the prediction of specific mortality categories would improve model discrimination and clinical interpretability. It is plausible that different clinical, laboratory, and perioperative predictors may be differentially associated with distinct causes of death, and that more specific endpoint definitions could strengthen predictive performance. However, such an approach would require adequately sized cause-specific outcome groups to ensure robust model development and validation.

Taken jointly, our findings demonstrate that accurate prediction of one-year mortality in elderly patients undergoing hip fracture surgery can be effectively achieved using both traditional ML and DL methods. Among the evaluated approaches, the manually developed XGB model delivered superior predictive performance. Notably, an automated ML workflow facilitated by an LLM achieved comparable results, highlighting the potential to democratise predictive analytics for clinical practitioners without advanced technical expertise. The integration of such predictive tools into routine perioperative clinical practice represents a promising avenue for enhancing risk stratification, informing clinical decision-making, and ultimately improving patient outcomes in this vulnerable population.

## Data Availability

The raw data supporting the conclusions of this article will be made available by the authors, without undue reservation.
