# Supplementary material for: Comparing manual vs. automated machine learning and deep learning models for predicting one-year mortality in elderly hip fracture patients
Source: Front Med (Lausanne). 2026 Jun 1;13:1804645. doi: 10.3389/fmed.2026.1804645 (PMC13265318; doi:10.3389/fmed.2026.1804645)
Supplement: Supplementary file 1 [file Table_1.DOCX]

import pandas as pd

file_path = 'mortality_1year_new.xlsx'

data = pd.read_excel(file_path)

missing_threshold = 0.2

columns_to_drop = data.columns[data.isnull().mean() > missing_threshold]

data_cleaned = data.drop(columns=columns_to_drop)

# Separate continuous and discrete features

continuous_features = data_cleaned.select_dtypes(include=['float64', 'int64']).columns

categorical_features = data_cleaned.select_dtypes(include=['object']).columns

# Initialize summary lists

summary = []

# Continuous features

for feature in continuous_features:

stats = {

"Symbol": feature,

"N": data_cleaned[feature].notnull().sum(),

"Range": f"{data_cleaned[feature].min()} - {data_cleaned[feature].max()}",

"Mean": round(data_cleaned[feature].mean(), 2),

"SD": round(data_cleaned[feature].std(), 2)

}

summary.append(stats)

# Discrete features

for feature in categorical_features:

distribution = data_cleaned[feature].value_counts(normalize=True).to_dict()

stats = {

"Symbol": feature,

"N": data_cleaned[feature].notnull().sum(),

"Range": "-", # Not applicable for categorical variables

"Mean": "-", # Not applicable for categorical variables

"SD": "-", # Not applicable for categorical variables

"Distribution": distribution

}

summary.append(stats)

# Convert summary to a DataFrame for presentation

summary_df = pd.DataFrame(summary)

# Save to CSV for further inspection

summary_df.to_csv('Table1.csv', index=False)

summary_df
