# Supplementary material for: Comparing manual vs. automated machine learning and deep learning models for predicting one-year mortality in elderly hip fracture patients
Source: Front Med (Lausanne). 2026 Jun 1;13:1804645. doi: 10.3389/fmed.2026.1804645 (PMC13265318; doi:10.3389/fmed.2026.1804645)
Supplement: Supplementary file 2 [file Table_2.DOCX]

Supplemental Material A. Structured prompt provided to LLM for automated ML pipeline development

| *You are an advanced AI assistant tasked with developing an ML model for a clinical prediction task. The goal is to create a Python script that implements the necessary steps for data analysis, model development, and evaluation in a user-friendly and reproducible manner, specifically for non-ML experts. The dataset consists of clinical variables used to predict one-year mortality and includes both continuous and categorical features. The data is imbalanced, with the positive class (mortality) underrepresented. The dataset provided to you, is in the attached file. The dataset should be split into training (80%) and validation (20%) cohorts, stratified to maintain similar age and gender distributions. A 5-fold cross-validation procedure should be applied to the training cohort to ensure robust performance estimates. To address the class imbalance, implement the Synthetic Minority Oversampling Technique (SMOTE) to augment the training data, and evaluate models trained on both the original and SMOTE-balanced datasets to assess the impact on performance. To find the ML model, use the TPOT automatic ML framework. Use the file provided.*  *The evaluation process should use metrics such as accuracy, F1 score, recall, precision, false positive rate (FPR), true negative rate (TNR), and area under the Receiver Operating Characteristic curve (AUC). Feature importance should be analyzed using permutation importance and SHAP. Use the supplied table i uploaded*  *The output should include four elements. First, a table with all evaluation metrics for both the training and validation cohorts. Second, feature distribution as a bar chart (normalize the y-axis between 0 and 1 and order the features from high to low). Third, plot the AUC-ROC curve, add a random guess line. Finally, a SHAP figure. Clear documentation and user-friendly explanations are essential to ensure that the results are accessible to non-ML experts. All code should be compatible with Python 3.11. make sure you use the file provided, the columns include the features and the target is outcome_death_1y* |
| --- |

Supplemental Material B. The grid search’s value space used for each of the manually explored models. The ranges were picked to balance between computation time and the robustness of the optimization process.

| **Model** | **Grid search** |
| --- | --- |
| LR | C: [0.01, 0.1, 1, 10]  penalty: [l2, l1] |
| NB |  |
| KNN | n_neighbors: [3, 5, 7, 10, 20]  weights [uniform, distance] |
| SVM | C: [0.1, 1, 10]  kernel: [linear, rbf] |
| DT | max_depth: [3, 5, 7, 10], min_samples_split: [2, 5, 10] |
| RF | n_estimators: [50, 100, 200]  max_depth: [3, 5, 7, 9, 11, 13, 15, 17]  min_samples_split: [5, 10, 15]  min_samples_leaf: [2, 4, 6]  max_features: [sqrt, log2] |
| XGB | n_estimators: [50, 100, 200]  max_depth: [3, 5, 7, 9, 11, 13, 15, 17]  learning_rate: [0.01, 0.05, 0.1]  subsample: [0.8, 0.9]  colsample_bytree: [0.8, 0.9]  reg_alpha: [0, 0.1, 1]  reg_lambda: [0, 0.1, 1] |
| MLP | hidden_layer_sizes: [(50,), (100,), (50, 50), (100, 50)]  alpha: [0.0001, 0.001, 0.01, 0.1]  learning_rate: [constant, adaptive]  activation: [relu, tanh] |
| Tabnet | n_d: [8, 16, 24]  n_a: [8, 16, 24]  n_steps: [3, 5]  gamma: [1.2, 1.5] |
| TT | TabTransformerWrapper( categories=[3, 3, 3, 3, 3, 3, 3, 3, 3]  num_continuous=89, dim=32,  depth=6, heads=8, attn_dropout=0.1, ff_dropout=0.1, epochs=10, lr=0.001),  params: { dim: [16, 32, 64],  depth: [4, 6, 8],  heads: [4, 8],  epochs: [10, 20] } |

Supplemental Table 1. A table of the independent variables used in developing the prediction model.

| **Variables** | **Unit of measurement** | **Measurement timing** |
| --- | --- | --- |
| age | years | Baseline |
| sex | category (M/F) | Baseline |
| country_of_birth | country | Baseline |
| height | cm | Baseline |
| weight | kg | Baseline |
| bmi | kg/m² | Baseline |
| smoking | binary (Yes/No) | Baseline |
| alcohol | binary (Yes/No) | Baseline |
| ubnormal_sleep | binary (Yes/No) | Baseline |
| cognition | categorical | Baseline |
| consciousness | categorical | Baseline |
| functional_status | categorical | Baseline |
| previous_hospitalization_6mo | binary (Yes/No) | Baseline |
| cohabilitation | categorical | Baseline |
| mobility | categorical | Baseline |
| time_trauma_surgery_less24h | binary (Yes/No) | Baseline |
| time_trauma_surgery_less48h | binary (Yes/No) | Baseline |
| time_trauma_surgery_more48h | binary (Yes/No) | Baseline |
| er_time_h | hours | ER admission |
| er_pnb | binary (Yes/No) | ER admission |
| baseline_creatinine | mg/dL | Baseline |
| baseline_urea | mg/dL | Baseline |
| baseline_sodium | mmol/L | Baseline |
| baseline_potassium | mmol/L | Baseline |
| baseline_lactate | mmol/L | Baseline |
| baseline_ph | pH | Baseline |
| baseline_hco3 | mmol/L | Baseline |
| baseline_base_excess | mmol/L | Baseline |
| baseline_troponin | ng/mL | Baseline |
| baseline_crp | mg/L | Baseline |
| baseline_hb | g/dL | Baseline |
| baseline_wbc | ×10³/µL | Baseline |
| baseline_lymph | % | Baseline |
| baseline_plt | ×10³/µL | Baseline |
| baseline_albumin | g/dL | Baseline |
| baseline_glucose | mg/dL | Baseline |
| baseline_inr | ratio | Baseline |
| baseline_ptt | seconds | Baseline |
| baseline_fib | mg/dL | Baseline |
| ejection_fraction | % | Baseline |
| as_ava_moderate | binary (Yes/No) | Baseline |
| as_ava_severe_critical | binary (Yes/No) | Baseline |
| as_pressuregradient_moderate | binary (Yes/No) | Baseline |
| as_pressuregradient_severe_critical | binary (Yes/No) | Baseline |
| aortic_valve_summary | categorical | Baseline |
| rv_function | categorical | Baseline |
| spo2_dept_first_less93 | binary (Yes/No) | Department arrival |
| dbp_dept_first_less60 | binary (Yes/No) | Department arrival |
| dbp_dept_first_more_110 | binary (Yes/No) | Department arrival |
| sbp_dept_first_less90 | binary (Yes/No) | Department arrival |
| sbp_dept_first_more180 | binary (Yes/No) | Department arrival |
| spo2_dept_last_less93 | binary (Yes/No) | Department departure |
| dbp_dept_last_less60 | binary (Yes/No) | Department departure |
| dbp_dept_last_more110 | binary (Yes/No) | Department departure |
| sbp_dept_last_less90 | binary (Yes/No) | Department departure |
| sbp_dept_last_more180 | binary (Yes/No) | Department departure |
| temp_dept_first_less36 | binary (Yes/No) | Department arrival |
| temp_dept_first_more37.5 | binary (Yes/No) | Department arrival |
| temp_dept_last_less36 | binary (Yes/No) | Department departure |
| temp_dept_last_more37.5 | binary (Yes/No) | Department departure |
| urinary_catheter | binary (Yes/No) | Perioperative |
| opioids_in_dept | binary (Yes/No) | Department |
| number_of_diseases | number | Baseline |
| number_of_chronic_medications | number | Baseline |
| procedure_cpt | CPT code | Surgery |
| or_time_min | minutes | Perioperative |
| surgery_time_min | minutes | Perioperative |
| surgery_room | identifier | Perioperative |
| asa | ASA classification | Baseline |
| anesthesia_type | categorical | Perioperative |
| aw_mgmt | categorical | Perioperative |
| inhalational_anaesthesia | binary (Yes/No) | Perioperative |
| mac_mean | MAC | Intraoperative |
| intraop_hypotermia | binary (Yes/No) | Intraoperative |
| IOH55 | binary (Yes/No) | Intraoperative |
| IOH60 | binary (Yes/No) | Intraoperative |
| IOH65 | binary (Yes/No) | Intraoperative |
| intraop_blood | binary (Yes/No) | Intraoperative |
| preop_blood | binary (Yes/No) | Preoperative |
| intraop_morphine_dose | mg/kg | Intraoperative |
| intraop_ephedrine | mg/kg | Intraoperative |
| intraop_phenylephrine | mcg/kg | Intraoperative |
| intraop_midazolam | mg/kg | Intraoperative |
| intraop_fentanyl_dose | mcg/kg | Intraoperative |
| neostigmine | binary (Yes/No) | Intraoperative |
| intraop_n2o | binary (Yes/No) | Intraoperative |
| intraop_ketamine | mg/kg | Intraoperative |
| intraop_ketorolac | mg/kg | Intraoperative |
| intraop_optalgin | mg/kg | Intraoperative |
| intraop_meperedine | mg/kg | Intraoperative |
| spinal_fentanyl_mcg | mcg/kg | Intraoperative |
| intraop_paracetamol | mg/kg | Intraoperative |
| paracetamol_before_incision | binary (Yes/No) | Intraoperative |
| intraop_tramadol | mg/kg | Intraoperative |
| intraop_antifibrinolytic | binary (Yes/No) | Intraoperative |
| intraop_aline | binary (Yes/No) | Intraoperative |
| intraop_cvc | binary (Yes/No) | Intraoperative |
| pacu_time_min | minutes | PACU |
